# Effectiveness of mRNA Vaccination in Preventing COVID-19–Associated Invasive Mechanical Ventilation and Death — United States, March 2021–January 2022

**DOI:** 10.15585/mmwr.mm7112e1

**Published:** 2022-03-25

**Authors:** Mark W. Tenforde, Wesley H. Self, Manjusha Gaglani, Adit A. Ginde, David J. Douin, H. Keipp Talbot, Jonathan D. Casey, Nicholas M. Mohr, Anne Zepeski, Tresa McNeal, Shekhar Ghamande, Kevin W. Gibbs, D. Clark Files, David N. Hager, Arber Shehu, Matthew E. Prekker, Anne E. Frosch, Michelle N. Gong, Amira Mohamed, Nicholas J. Johnson, Vasisht Srinivasan, Jay S. Steingrub, Ithan D. Peltan, Samuel M. Brown, Emily T. Martin, Arnold S. Monto, Akram Khan, Catherine L. Hough, Laurence W. Busse, Abhijit Duggal, Jennifer G. Wilson, Nida Qadir, Steven Y. Chang, Christopher Mallow, Carolina Rivas, Hilary M. Babcock, Jennie H. Kwon, Matthew C. Exline, Mena Botros, Adam S. Lauring, Nathan I. Shapiro, Natasha Halasa, James D. Chappell, Carlos G. Grijalva, Todd W. Rice, Ian D. Jones, William B. Stubblefield, Adrienne Baughman, Kelsey N. Womack, Jillian P. Rhoads, Christopher J. Lindsell, Kimberly W. Hart, Yuwei Zhu, Katherine Adams, Diya Surie, Meredith L. McMorrow, Manish M. Patel, IVY Network

**Affiliations:** ^1^CDC COVID-19 Emergency Response Team; ^2^Vanderbilt University Medical Center, Nashville, Tennessee; ^3^Baylor Scott & White Health, Temple, Texas; ^4^Texas A&M University College of Medicine, Temple, Texas; ^5^University of Colorado School of Medicine, Aurora, Colorado; ^6^University of Iowa, Iowa City, Iowa; ^7^Wake Forest University Baptist Medical Center, Winston-Salem, North Carolina; ^8^Johns Hopkins Hospital, Baltimore, Maryland; ^9^Hennepin County Medical Center, Minneapolis, Minnesota; ^10^Montefiore Healthcare Center, Albert Einstein College of Medicine, Bronx, New York; ^11^University of Washington School of Medicine, Seattle, Washington; ^12^Baystate Medical Center, Springfield, Massachusetts; ^13^Intermountain Medical Center, Salt Lake City, Utah; ^14^University of Utah, Salt Lake City, Utah; ^15^University of Michigan School of Public Health, Ann Arbor, Michigan; ^16^Oregon Health & Science University Hospital, Portland, Oregon; ^17^Emory University School of Medicine, Atlanta, Georgia; ^18^Cleveland Clinic, Cleveland, Ohio; ^19^Stanford University School of Medicine, Palo Alto, California; ^20^Ronald Reagan-UCLA Medical Center, Los Angeles, California; ^21^University of Miami, Miami, Florida; ^22^Washington University, St. Louis, Missouri; ^23^Ohio State University Wexner Medical Center, Columbus, Ohio; ^24^University of Michigan School of Medicine, Ann Arbor, Michigan; ^25^Beth Israel Deaconess Medical Center, Boston, Massachusetts.

COVID-19 mRNA vaccines (BNT162b2 [Pfizer-BioNTech] and mRNA-1273 [Moderna]) are effective at preventing COVID-19–associated hospitalization (*1*–*3*). However, how well mRNA vaccines protect against the most severe outcomes of these hospitalizations, including invasive mechanical ventilation (IMV) or death is uncertain. Using a case-control design, mRNA vaccine effectiveness (VE) against COVID-19–associated IMV and in-hospital death was evaluated among adults aged ≥18 years hospitalized at 21 U.S. medical centers during March 11, 2021–January 24, 2022. During this period, the most commonly circulating variants of SARS-CoV-2, the virus that causes COVID-19, were B.1.1.7 (Alpha), B.1.617.2 (Delta), and B.1.1.529 (Omicron). Previous vaccination (2 or 3 versus 0 vaccine doses before illness onset) in prospectively enrolled COVID-19 case-patients who received IMV or died within 28 days of hospitalization was compared with that among hospitalized control patients without COVID-19. Among 1,440 COVID-19 case-patients who received IMV or died, 307 (21%) had received 2 or 3 vaccine doses before illness onset. Among 6,104 control-patients, 4,020 (66%) had received 2 or 3 vaccine doses. Among the 1,440 case-patients who received IMV or died, those who were vaccinated were older (median age = 69 years), more likely to be immunocompromised* (40%), and had more chronic medical conditions compared with unvaccinated case-patients (median age = 55 years; immunocompromised = 10%; p<0.001 for both). VE against IMV or in-hospital death was 90% (95% CI = 88%–91%) overall, including 88% (95% CI = 86%–90%) for 2 doses and 94% (95% CI = 91%–96%) for 3 doses, and 94% (95% CI = 88%–97%) for 3 doses during the Omicron-predominant period. COVID-19 mRNA vaccines are highly effective in preventing COVID-19–associated death and respiratory failure treated with IMV. CDC recommends that all persons eligible for vaccination get vaccinated and stay up to date with COVID-19 vaccination (*4*).

Using surveillance data from the Influenza and Other Viruses in the Acutely Ill (IVY) Network, a case-control analysis was conducted to evaluate effectiveness of mRNA COVID-19 vaccines against COVID-19–associated IMV or death. During March 11, 2021–January 24, 2022, adults aged ≥18 years hospitalized at 21 medical centers in 18 states^†^ who received testing for SARS-CoV-2 were enrolled. Case-patients were adults who were hospitalized with COVID-19–like illness^§^ and who received positive SARS-CoV-2 nucleic acid amplification test (NAAT) or antigen test results within 10 days of illness onset. Case-patients in this analysis were limited to those who received IMV or died in the hospital within 28 days of admission. Control-patients were hospitalized adults with or without COVID-19–like illness who received a negative NAAT test result for SARS-CoV-2 within 10 days of illness onset. Individual matching was not performed, but sites attempted 1:1 enrollment of case-patients and controls, with controls enrolled within 2 weeks of case-patients. Patients or their proxies were interviewed about demographic and clinical characteristics and COVID-19 vaccination history. COVID-19 mRNA vaccination status (i.e., receipt of Pfizer-BioNTech or Moderna vaccine products) was ascertained from state registry data, hospital electronic medical records, vaccination record cards, and self-report. For this analysis, patients were included if they 1) received 2 doses of an mRNA vaccine, with the second dose administered ≥14 days before illness onset, 2) received 3 doses of an mRNA vaccine following authorization^¶^ with the third dose administered ≥7 days before illness onset, or 3) received no COVID-19 mRNA vaccine doses before illness onset. Information about chronic medical conditions and in-hospital outcomes, including IMV or death within 28 days of admission, were collected through structured chart reviews. 

Differences in demographic and clinical characteristics between COVID-19 case-patients who were vaccinated with 2 or 3 vaccine doses versus unvaccinated were compared using Pearson’s chi-square for categorical variables or Wilcoxon rank-sum tests for continuous variables. VE was calculated using unconditional logistic regression by comparing the odds for previous mRNA vaccination (2 or 3 doses) among COVID-19 case-patients who received IMV or experienced in-hospital death versus control-patients. VE was calculated as (1 − odds ratio) × 100, and estimates were adjusted for U.S. Health and Human Services region, calendar time in biweekly intervals, age, sex, and self-reported race and Hispanic ethnicity as prespecified covariates. Results were stratified by age, immunocompromising conditions, number of categories of chronic medical conditions,** number of COVID-19 mRNA vaccine doses received, and variant-predominant period when admitted to hospital. Variant-predominant periods were defined as pre-Delta (March 11–July 3, 2021), Delta (July 4–December 25, 2021), or Omicron (December 26, 2021–January 24, 2022), based on when a variant accounted for >50% of sequenced SARS-CoV2 viruses using on whole-genome sequencing of specimens collected in the IVY network. An additional sensitivity analysis was conducted by restricting COVID-19–negative controls to those known to have received IMV or to have died in the hospital within 28 days of admission. Analyses were conducted using STATA software (version 16.0; StataCorp); p-values <0.05 were considered statistically significant. This activity was determined to be public health surveillance by each participating site and CDC and was conducted in a manner consistent with applicable federal law and CDC policy.^††^

Among 9,211 COVID-19 case-patients with IMV or in-hospital death and COVID-19–negative controls enrolled during March 11, 2021–January 24, 2022, 1,667 (18%) were excluded from the analysis. The most common reasons for exclusion included receiving a licensed mRNA COVID-19 vaccine but not being in a vaccination group considered in this analysis (638), receiving a non-mRNA COVID-19 vaccine product (445), inability to determine vaccination status (279), COVID-19–like illness onset after hospital admission (119), and receiving a third vaccine dose before authorization (96); 90 patients were excluded for other reasons. Among 7,544 included patients, 1,440 (19%) were COVID-19 case-patients with IMV, death, or both, and 6,104 (81%) were COVID-19–negative controls. Compared with unvaccinated case-patients with IMV or in-hospital death, those who were vaccinated (2 or 3 doses) were older (median age 69 versus 55 years; p<0.001), more likely to live in a long-term care facility (11% versus 2%; p<0.001), more likely to have been hospitalized previously in the past year (44% versus 22%; p<0.001), more likely to have immunocompromising conditions (40% versus 10%; p<0.001), and had more chronic medical conditions (Table 1). 

**TABLE 1:** Characteristics of case-patients with laboratory-confirmed COVID-19 who received invasive mechanical ventilation or died in the hospital (n = 1,440) and COVID-19 test-negative controls, by mRNA vaccination group — 21 hospitals,* 18 states, March 2021–January 2022

Characteristic	COVID-19 test-negative controls, no. (%) (n = 6,104)	Case patients with IMV or death, no. (%)		P-value^†^
		Vaccinated (n = 307)	Unvaccinated (n = 1,133)	
**Age, median, yrs (IQR)**	63 (50–72)	69 (60–77)	55 (42–66)	<0.001
**Female sex**	3,043 (49.9)	135 (44.0)	463 (40.9)	0.327
**Race and ethnicity^§^**				
White, non-Hispanic	3,690 (60.5)	191 (62.2)	638 (56.3)	0.317
Black, non-Hispanic	1,276 (20.9)	49 (16.0)	200 (17.7)	
Hispanic	792 (13.0)	47 (15.3)	200 (17.7)	
All other races, non-Hispanic	262 (4.3)	15 (4.9)	59 (5.2)	
Unknown	84 (1.4)	5 (1.6)	36 (3.2)	
**LTCF resident,^¶^ no./total no. (%)**	330/5,920 (5.6)	32/284 (11.3)	20/1,023 (2.0)	<0.001
**One or more previous hospitalizations in the last year, no./total no. (%)**	3,097/5,674 (54.6)	125/284 (44.0)	217/975 (22.3)	<0.001
**Current tobacco use, no./total no. (%)**	1,035/5,426 (19.1)	25/241 (10.4)	97/835 (11.6)	0.592
**Immunocompromising condition, no./total no.**	1,504 (24.6)	123 (40.1)	109 (9.6)	<0.001
**Among immunocompetent, no. of chronic medical condition types, median (IQR)**	2 (1–3)	2 (1.5–3)	1 (0–2)	<0.001
**Specific categories of conditions**				
Chronic cardiovascular disease	4,246 (69.6)	252 (82.1)	571 (50.4)	<0.001
Chronic pulmonary disease	2,016 (33.0)	91 (29.6)	213 (18.8)	<0.001
Diabetes mellitus	1,991 (32.6)	140 (45.6)	323 (28.5)	<0.001
**Received 2 or 3 mRNA vaccine doses**	4,020 (65.9)	307 (100)	0 (—)	—
**Vaccinated, no. of doses received**				
2	3,488 (86.8)	277 (90.2)	—	—
3	532 (13.2)	30 (9.8)	—	—

**Abbreviations:** IMV = invasive mechanical ventilation; LTCF = long-term care facility.

* Hospitals (cities, states) included Baystate Medical Center (Springfield, Massachusetts), Beth Israel Deaconess Medical Center (Boston, Massachusetts), Montefiore Medical Center (Bronx, New York), Vanderbilt University Medical Center (Nashville, Tennessee), University of Miami Medical Center (Miami, Florida), Emory University Medical Center (Atlanta, Georgia), Johns Hopkins Hospital (Baltimore, Maryland), Wake Forest University Baptist Medical Center (Winston-Salem, North Carolina), Baylor Scott & White Health (Temple, Texas), University of Iowa Hospitals (Iowa City, Iowa), University of Michigan Hospital (Ann Arbor, Michigan), Hennepin County Medical Center (Minneapolis, Minnesota), Barnes-Jewish Hospital (St. Louis, Missouri), Cleveland Clinic (Cleveland, Ohio), Ohio State University Wexner Medical Center (Columbus, Ohio), Stanford University Medical Center (Stanford, California), UCLA Medical Center (Los Angeles, California), UCHealth University of Colorado Hospital (Aurora, Colorado), Oregon Health & Science University Hospital (Portland, Oregon), Intermountain Medical Center (Murray, Utah), and University of Washington (Seattle, Washington).

^†^ Comparisons between vaccinated and unvaccinated COVID-19 case-patients made by Pearson’s chi-square test for categorical variables or Wilcoxon rank-sum test for continuous variables.

^§^ Race and ethnic groups were self-reported as a single category by patient or proxy listed in table; “All other races, non-Hispanic” included Asian (151), Native American or Alaska Native (52), Native Hawaiian or other Pacific Islander (33), and Other (100).

^¶^ LTCF included residence in a nursing home, assisted living home, or rehab hospital/other subacute or chronic facility before hospital admission.

Overall VE against COVID-19–associated IMV or death across the surveillance period was 90% (95% CI = 88%–91%) (Table 2), similar to that for IMV only (91%; 95% CI = 89%–92%) and in-hospital death only (88%; 95% CI = 85%–90%), and similar in a sensitivity analysis restricting COVID-19 test-negative control-patients to those who also received IMV or died in the hospital (86%; 95% CI = 82%–89%). Among recipients of 2 vaccine doses, VE over the entire study period was 92% (95% CI = 90%–94%) at 14–150 days after receipt of the second dose versus 84% (95% CI = 80%–87%) at >150 days postvaccination. VE was 94% (95% CI = 91%–96%) among recipients of 3 vaccine doses. Among immunocompetent adults with no chronic medical conditions, VE for 2 or 3 vaccine doses was 98% (95% CI = 97%–99%). VE was lowest among adults with immunocompromising conditions (74%; 95% CI = 64%–81%). However, among 123 vaccinated COVID-19 case-patients with immunocompromising conditions, only 17 (14%) had received 3 vaccine doses and were considered fully vaccinated.^§§^ During the Omicron period, VE against IMV or in-hospital death was 79% (95% CI = 66%–87%) for recipients of 2 doses and 94% (95% CI = 88%–97%) for recipients of 3 doses.

**TABLE 2:** Effectiveness of COVID-19 mRNA vaccines against COVID-19–associated invasive mechanical ventilation or in-hospital death — 21 hospitals, 18 states,*^,†^ March 2021–January 2022

Group/Characteristic	No. of vaccinated case-patients with IMV or death/total no. of case-patients (%)	No. of vaccinated control-patients/ total no. of control-patients (%)	Vaccine effectiveness, % (95% CI)
**All variant periods^§^**	307/1,440 (21.3)	4,020/6,104 (65.9)	90 (88–91)
**No. of mRNA vaccine doses received**			
2	277/1,410 (19.6)	3,488/5,572 (62.6)	88 (86–90)
14–150 days after dose 2	92/1,225 (7.5)	2,039/4,123 (49.5)	92 (90–94)
>150 days after dose 2	185/1,318 (14.0)	1,449/3,533 (41.0)	84 (80–87)
3	30/1,163 (2.6)	532/2,616 (20.3)	94 (91–96)
**Age group, yrs**			
18–64	115/931 (12.4)	1,807/3,326 (54.3)	91 (89–93)
≥65	192/509 (37.7)	2,213/2,778 (79.7)	88 (84–90)
**Health status**			
Immunocompromised	123/232 (53.0)	1,090/1,504 (72.5)	74 (64–81)
Immunocompetent	184/1,208 (15.2)	2,930/4,600 (63.7)	92 (91–94)
**No. of chronic conditions among immunocompetent**			
None	12/368 (3.3)	322/642 (50.2)	98 (97–99)
1	34/337 (10.1)	647/1,094 (59.1)	95 (92–96)
2	60/264 (22.7)	886/1,320 (67.1)	89 (85–93)
≥3	78/239 (32.6)	1,075/1,544 (69.6)	84 (78–89)
**Variant period,^¶^ no. of doses**			
**Pre-Delta, 2 doses**	13/259 (5.0)	893/1,738 (51.4)	95 (90–97)
**Delta, 2 or 3 doses**	235/1,027 (22.9)	2,741/3,865 (70.9)	89 (87–91)
2 doses, median = 159 days after dose 2	218/1,010 (21.6)	2,402/3,526 (68.1)	88 (86–90)
3 doses, median = 35 days after dose 3	17/809 (2.1)	339/1,463 (23.2)	95 (91–97)
**Omicron, 2 or 3 doses**	59/154 (38.3)	386/501 (77.0)	86 (79–91)
2 doses, median = 256 days after dose 2	46/141 (32.6)	193/308 (62.7)	79 (66–87)
3 doses, median = 60 days after dose 3	13/108 (12.0)	193/308 (62.7)	94 (88–97)

**Abbreviations:** IMV = invasive mechanical ventilation; VE = vaccine effectiveness.

* Reported VE results are for 2 or 3 vaccine doses except where otherwise noted. VE was estimated using logistic regression comparing the odds of being vaccinated with 2 or 3 doses of an mRNA vaccine versus being unvaccinated for laboratory-confirmed cases with IMV or death and test-negative controls and calculated as VE = 100 × (1 − odds ratio). Logistic regression models were adjusted for date of hospital admission (biweekly intervals), U.S. Department of Health and Human Services region of hospital (10 regions), age group (18–49, 50–64, and ≥65 years), sex, and race/ethnicity (non-Hispanic White, non-Hispanic Black, Hispanic of any race, non-Hispanic other, or unknown). Age-specific models were adjusted for age as a continuous variable.

^†^ Hospitals (cities, states) included Baystate Medical Center (Springfield, Massachusetts), Beth Israel Deaconess Medical Center (Boston, Massachusetts), Montefiore Medical Center (Bronx, New York), Vanderbilt University Medical Center (Nashville, Tennessee), University of Miami Medical Center (Miami, Florida), Emory University Medical Center (Atlanta, Georgia), Johns Hopkins Hospital (Baltimore, Maryland), Wake Forest University Baptist Medical Center (Winston-Salem, North Carolina), Baylor Scott & White Health (Temple, Texas), University of Iowa Hospitals (Iowa City, Iowa), University of Michigan Hospital (Ann Arbor, Michigan), Hennepin County Medical Center (Minneapolis, Minnesota), Barnes-Jewish Hospital (St. Louis, Missouri), Cleveland Clinic (Cleveland, Ohio), Ohio State University Wexner Medical Center (Columbus, Ohio), Stanford University Medical Center (Stanford, California), UCLA Medical Center (Los Angeles, California), UCHealth University of Colorado Hospital (Aurora, Colorado), Oregon Health & Science University Hospital (Portland, Oregon), Intermountain Medical Center (Murray, Utah), and University of Washington (Seattle, Washington).

^§^ With vaccination defined as receipt of either 2 or 3 mRNA vaccine doses.

^¶^ Variant periods were defined by hospital admission dates as the following: pre-Delta (when the Alpha variant dominated but other variants co-circulated), March 11–July 3, 2021; Delta, July 4–December 25, 2021, and Omicron, December 26, 2021–January 24, 2022. Start dates for variant periods were selected based on calendar weeks during which the variant accounted for >50% of sequenced viruses that had lineage determination from whole-genome sequencing.

## Discussion

Analysis of data on severe COVID-19 outcomes from a multistate hospital network found that receipt of 2 or 3 doses of a COVID-19 mRNA vaccine conferred 90% protection against COVID-19–associated IMV or in-hospital death among adults. Most vaccinated patients who experienced COVID-19–associated IMV or who died in hospital were older or had complex underlying conditions, commonly immunosuppression. Protection against IMV or death was consistent throughout the Delta and Omicron periods and was higher in adults who received a third vaccine dose, including 94% during the Omicron period. These findings reinforce the highly protective effects of up-to-date COVID-19 vaccination against severe illness and death among adults, including against current SARS-CoV-2 variants.

SARS-CoV-2 infection, like that from other respiratory viruses, is manifested by a gradient in illness severity, ranging from asymptomatic or mild infection to critical or fatal complications (*2*,*5*). Protection against asymptomatic or milder infection might be reduced by waning of neutralizing antibody levels after vaccination or by immune evasion by emerging variants (*6*,*7*). However, vaccination stimulates long-lasting memory B and T-cell responses that might limit severity of illness in infected adults (*8*). Some studies have found that COVID-19 vaccines provided reduced protection against milder infection (*6*,*7*). The findings of this study indicate that COVID-19 vaccines provide strong protection against severe COVID-19 resulting in respiratory failure or in-hospital death.

The findings in this report are subject to at least five limitations. First, although receipt of 3 mRNA vaccine doses was associated with better protection against critical COVID-19 outcomes than was receipt of 2 doses, understanding the durability of protection over time or against emerging SARS-CoV-2 variants will require ongoing surveillance. Second, although adjustments were made for calendar time, age, and race/ethnicity, among other potential confounders, unmeasured or residual confounding is possible. Third, control-patients hospitalized without COVID-19 might not have been fully representative of case-patients likely to receive IMV or die while in the hospital. In a sensitivity analysis restricting control-patients to those who received IMV or died from causes not related to COVID-19, results were similar. Fourth, although representing 18 states, patients in this study might not be entirely representative of the general U.S. adult population. Most hospitalized patients had multiple chronic medical conditions, and the overall VE observed in this analysis might underestimate protection in healthier populations. VE against COVID-19–associated IMV or in-hospital death in adults without chronic medical conditions was highest at 98%. Finally, although VE was lower for adults with immunocompromising conditions, most of these persons had not received the third mRNA vaccine dose recommended as part of a primary vaccine series for immunocompromised persons.

Through February 2022, nearly 1 million COVID-19–associated deaths have occurred in the United States, primarily in unvaccinated persons (*9*). COVID-19 vaccination is likely to prevent a majority of COVID-19–associated deaths and other life-threatening outcomes. CDC recommends that all persons eligible for vaccination get vaccinated and stay up to date with COVID-19 vaccination (*4*).

**Summary**

**What is already known about this topic?**

COVID-19 mRNA vaccines provide protection against COVID-19 hospitalization among adults. However, how well mRNA vaccines protect against the most severe outcomes of COVID-19–related illness, including use of invasive mechanical ventilation (IMV) or death, is uncertain.

**What is added by this report?**

Receiving 2 or 3 doses of an mRNA COVID-19 vaccine was associated with a 90% reduction in risk for COVID-19–associated IMV or death. Protection of 3 mRNA vaccine doses during the period of Omicron predominance was 94%.

**What are the implications for public health practice?**

COVID-19 mRNA vaccines are highly effective in preventing the most severe forms of COVID-19. CDC recommends that all persons eligible for vaccination get vaccinated and stay up to date with COVID-19 vaccination.

## Footnotes

* Immunocompromising conditions included having one or more of the following conditions: active solid organ cancer (active cancer defined as treatment for the cancer or newly diagnosed cancer in the past 6 months); active hematologic cancer (e.g., leukemia, lymphoma, or myeloma); HIV infection without AIDS; AIDS; congenital immunodeficiency syndrome; previous splenectomy; previous solid organ, stem cell, or bone marrow transplant; immunosuppressive medication; systemic lupus erythematosus; rheumatoid arthritis; psoriasis; scleroderma; or inflammatory bowel disease, including Crohn’s disease or ulcerative colitis.

† Hospitals (cities, states) included Baystate Medical Center (Springfield, Massachusetts), Beth Israel Deaconess Medical Center (Boston, Massachusetts), Montefiore Medical Center (Bronx, New York), Vanderbilt University Medical Center (Nashville, Tennessee), University of Miami Medical Center (Miami, Florida), Emory University Medical Center (Atlanta, Georgia), Johns Hopkins Hospital (Baltimore, Maryland), Wake Forest University Baptist Medical Center (Winston-Salem, North Carolina), Baylor Scott & White Health (Temple, Texas), University of Iowa Hospitals (Iowa City, Iowa), University of Michigan Hospital (Ann Arbor, Michigan), Hennepin County Medical Center (Minneapolis, Minnesota), Barnes-Jewish Hospital (St. Louis, Missouri), Cleveland Clinic (Cleveland, Ohio), Ohio State University Wexner Medical Center (Columbus, Ohio), Stanford University Medical Center (Stanford, California), UCLA Medical Center (Los Angeles, California), UCHealth University of Colorado Hospital (Aurora, Colorado), Oregon Health & Science University Hospital (Portland, Oregon), Intermountain Medical Center (Murray, Utah), and University of Washington (Seattle, Washington).

§ COVID-19–like illness was defined as having one or more of the following signs or symptoms: fever, cough, shortness of breath, loss of taste, loss of smell, use of respiratory support for the acute illness, or new pulmonary findings on chest imaging consistent with pneumonia.

¶ Recipients of 3 doses of mRNA vaccine were included if they received a third dose after Emergency Use Authorization (after August 12, 2021, for adults with immunocompromising conditions and after September 22, 2021, for adults without immunocompromising conditions) and they received the third dose ≥28 days after dose 2 to complete a primary vaccine series for adults with immunocompromising conditions and ≥150 days after dose 2 as a booster dose for adults without immunocompromising conditions.

** Categories of nonimmunocompromising chronic medical conditions included cardiovascular disease, neurologic disease, pulmonary disease, gastrointestinal disease, endocrine disease, renal disease, hematologic disease, and other conditions (e.g., unintentional weight loss of ≥10 pounds in the past 90 days, sarcoidosis, or amyloidosis).

†† 45 C.F.R. part 46.102(l)(2), 21 C.F.R. part 56; 42 U.S.C. Sect. 241(d); 5 U.S.C. Sect. 552a; 44 U.S.C. Sect. 3501 et seq.

§§ For adults with moderately to severely immunocompromising conditions who have received 2 doses of an mRNA vaccine, a third vaccine dose is recommended ≥28 days after the second dose as part of a primary vaccine series, with a fourth vaccine dose recommended ≥3 months later as a booster dose.
